# Simultaneous determination of methylcarbamate and ethylcarbamate in fermented foods and beverages by derivatization and GC-MS analysis

**DOI:** 10.1186/1752-153X-6-157

**Published:** 2012-12-13

**Authors:** Ho-Sang Shin, Eun-Young Yang

**Affiliations:** 1 Department of Environmental Education, Kongju, 314-701, Republic of Korea; 2 Department of Environmental Science, Kongju National University, Kongju, 314-701, Republic of Korea

**Keywords:** Methylcarbamate, Ethylcarbamate, Fermented food, Beverages, GC-MS

## Abstract

**Background:**

Methylcarbamate (MC) and ethylcarbamate (EC) are toxic compounds that commonly exist in fermented food and beverages. In order to estimate the risk for their exposure, a sensitive simultaneous analytical method is required

**Results:**

A simultaneous determination of MC and EC was described based on derivatization with 9-xanthydrol and consecutive detection using gas chromatography–mass spectrometry. The derivatization of MC and EC was performed directly in food or beverages and the reaction conditions were established through changing various parameters. The detection and the quantification limits were 0.01-0.03 μg/kg and 0.03-0.1 μg/kg, respectively, and the interday relative standard deviation was less than 12% at concentrations of 2.0 and 50 μg/kg. MC and EC were measured from 0.4 μg/kg to 85.8 μg/kg in sixteen Korean fermented foods and eleven beverages.

**Conclusion:**

A simple, sensitive method to detect MC and EC in several solid foods and liquid foods was developed based on derivatization with 9-xanthydrol for 10 min at an ambient temperature. The method may useful for routine analysis of MC and EC in numerous food samples.

## Background

Ethylcarbamate (EC, urethane, C_2_H_5_OCONH_2_) is a known genotoxic carcinogen that commonly exists in fermented food and beverages due to the natural biochemical processes in the fermentation process [1,2]. EC was re-classified as a carcinogen (Group 2A) by the International Agency for Research on Cancer (IARC) in 2007 [3] and has already been regulated in several countries such as Germany, USA, Canada, France and the Czech Republic [2]. A report from a commission by the European Food Safety Authority (EFSA) issued in 2010 [4] recommended that special attention should be paid to spirits distilled from stone fruits. Furthermore, EC has been detected in various fermented products such as bread, yoghurt, cheese, soy sauce, vinegar and alcoholic beverages [5,6].

Methylcarbamate (MC, methylurethane, CH_3_OCONH_2_) is simplest ester of carbamic acid. MC has a relatively low toxicity, otherwise, there is experimental evidence that MC is mutagenic in Droso phila [7] and carcinogenic in rats [8].

EC and MC can co-exist through natural formation during the fermentation processes [9]. In order to estimate the risk for EC and MC exposure, a sensitive simultaneous analytical method in fermented foods and beverages is required.

Many methods for detecting EC in beverages have been reported, such as high-performance liquid chromatography (HPLC) [10-12], liquid chromatography tandem mass spectrometry (LC-MS/MS) [13], gas chromatography (GC) [14-17], and gas chromatography mass spectrometry (GC–MS) [6,9,18-27].

Several assay methods have been based on headspace solid-phase micro extraction (HS-SPME) [14,15,22,28], where the headspace is discriminatory in nature because only the volatile compounds in the injection vials can be transferred to the GC system. Many volatile alcohols and interferences exist in fermented food and beverages, give much interference, and have a short fiber life time. Liquid-liquid extraction (LLE) [16,19,21] and solid phase extraction (SPE) [20,21,26] are often used to determine the EC content in alcoholic beverages. Although it is a traditional extraction technique, LLE represents a convenient method when it is connected with derivatization. Also, 9-xanthydrol has been used to improve the fluorescence of EC in the HPLC method [10-12] and to improve the sensitivity of EC using the GC-MS [21]. However, until now, analytical target compounds and matrices were limited to EC and liquid phases such as spirits or beverages. Another drawback with the methods is that EC is derivatized using 9-xanthydrol after extraction and concentration, and in this case volatile MC and EC can be lost during the evaporation process.

GC coupled with mass spectrometry (GC-MS) is the most widely used due to its good resolution, sensitivity and selectivity. Although the GC-MS methods are very selective and sensitive, it is difficult to detect to ng/kg levels without concentration and derivatization.

In this study, the derivatization parameters that enable the direct reaction of MC and EC in food or beverages are established. The xanthyl methylcarbamate or xanthyl ethylcarbamate derivatives that were formed were extracted by LLE and detected by GC–MS. Therefore, the experiment reported in this paper aimed to optimize the parameters of the derivatization, extraction and GC-MS detection in order to simultaneously determine the MC and EC in fermented foods and beverages, and in order to apply the modified method in the analysis of seventeen real samples.

## Experimental

### Materials

All organic solvents used were HPLC grade. Sodium chloride, potassium hydroxide, sodium bicarbonate, potassium carbonate, propanol, ethyl acetate, sodium sulfate, 9-xanthydrol (99%), methylcarbamate (98%), ethylcarbamate (EC, 99%), and butylcarbamate (98%) as internal standard were obtained from Sigma-Aldrich (St. Louis, MO, USA).

### Apparatus

All mass spectra were obtained with an Agilent 6891/5973N instrument (Agilent Technologies, Santa Clara, CA, USA). The ion source was operated in the electron ionization mode (EI; 70 eV). Full-scan mass spectra (m/z 45–600) were recorded in order to identify the analytes. An HP-5MS capillary column (60 m × 0.25 mm I.D. × 0.25 μm film thickness) was used. The samples were injected in the splitless mode. The flow rate of helium as a carrier gas was 0.6 mL/min. The injector temperature was set at 260°C. The oven temperature programs were set as follows. The initial temperature of 150°C was not held and increased to the first temperature hold of 210°C (held for 1 min) at 30°C/min, and then increased to the final temperature hold of 260°C (held for 4 min) at 10°C/min. The ions selected by SIM were m/z 222, 240 and 255 for xanthyl methylcarbamate, m/z 222, 240 and 269 for xanthyl ethylcarbamate and m/z 222, 240 and 297 for xanthyl butylcarbamate.

### Derivatization and extraction procedures

Fermented foods (soybean paste, red pepper paste and soy sauce) were purchased from several local markets or obtained from several homes. Beverages containing makgeolli (raw rice wine), soju (white distilled liquor), jeongjong (refined rice wine) and fruit liquor were purchased from several local markets.

A 2.0 g portion of each sample was homogenized for 10 min at 18,000 rpm in 5.0 mL of NaCl saturated solution using a homogenizer (PowerGen 125, Fisher Scientific, USA) after adding 80 μL of 0.1 M 9-xanthydrol solution in the propanol, 200 μL of 2.0 M HCl, and 20 μL of BC (2.5 mg/L in methanol). The derivatization reaction was conducted at an ambient temperature for 10 min in the dark, and then the solution was neutralized with 1.0 M KOH and the pH of the solution was controlled to 9.5 with 0.2 g of NaHCO_3_/K_2_CO_3_ (2:1, w/w). The solution was extracted twice with 5.0 mL of ethyl acetate. The organic layers were combined and dried by passing them through anhydrous sodium sulfate. The dried organic layer was then concentrated in a rotary evaporator (30°C, 300 mbar). The concentrated residue was dissolved in 100 μL of methanol and a 1.0 μL sample of the solution was injected into the GC-MS system.

The derivatization efficiencies were calculated at various temperatures (20, 30, 40, and 50°C), 9-xanthydrol amounts (20, 40, 60, 80, 100, and 120 μL of 0.1 M solution), heating times (5, 10, 15, 20, 30 and 60 min), and acid moralities (0.1, 0.2, 0.3, 0.4, 0.5 and 1.0 M). The pH of each sample was controlled with 2.0 M HCl. The optimum derivatization conditions of MC, EC and BC with 9-xanthydrol were determined using the amounts of the formed xanthyl methylcarbamate, xanthyl ethylcarbamate and xanthyl butylcarbamate.

### Calibration and quantification

The calibration curves for MC and EC were established through derivatizations after 1.0, 5.0, 20, 50, 100 and 200 ng of MC and EC standard solutions were added to 2.0 g of a control food (soybean paste), 5 mL of NaCl saturated solution, 20 μL of BC (2.5 mg/L in methanol), 80 μL of 0.1M 9-xanthydrol solution in propanol and 200 μL of 2.0 M HCl. The corresponding concentrations of the standards were 0.5, 2.5, 10, 25, 50 and 100 μg/kg. The ions selected for quantification were m/z 255 for xanthyl methylcarbamate, and m/z 240 for xanthyl ethylcarbamate and xanthyl butylcarbamate. The ratio of the peak area of the standard solution to that of the internal standard was used to quantify the compound.

## Results and discussion

### Optimization of the derivatization conditions in samples

**Figure 1:**
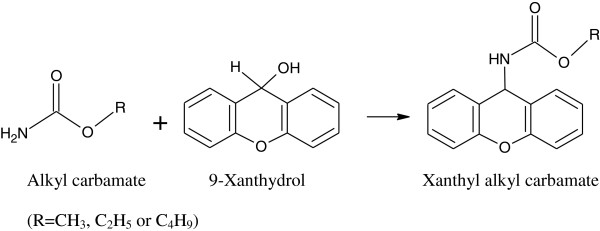
The reaction of alkyl carbamates with 9-xanthydrol.

The amino groups of MC, EC, and BC undertook the substitution reaction with 9-xanthydrol under acidic conditions in order to produce xanthyl methylcarbamate, xanthyl ethylcarbamate, and xanthyl butylcarbamate as shown in Figure 1, and it was possible to directly analyze the product by the GC-MS.

**Figure 2:**
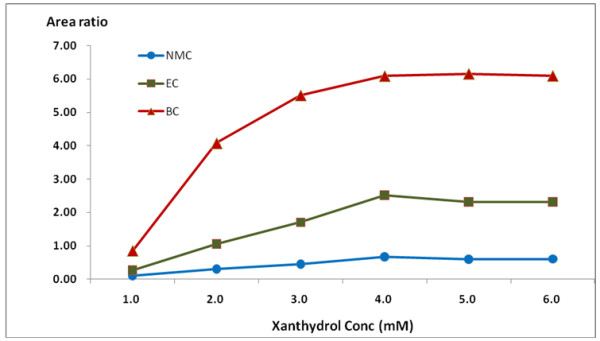
**Reaction yield of MC, EC and BC in relation to the amount of 9-xanthydrol.** (This experiment was performed at a reaction time of 10 min and a reaction temperature of 20°C).

**Figure 3:**
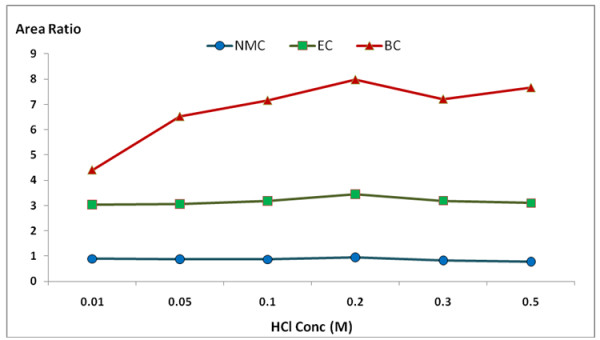
**Effect of HCl concentration on the reaction of MC, EC and BC with 9-xanthydrol.** (This experiment was performed at a reaction time of 10 min and a reaction temperature of 20°C).

**Figure 4:**
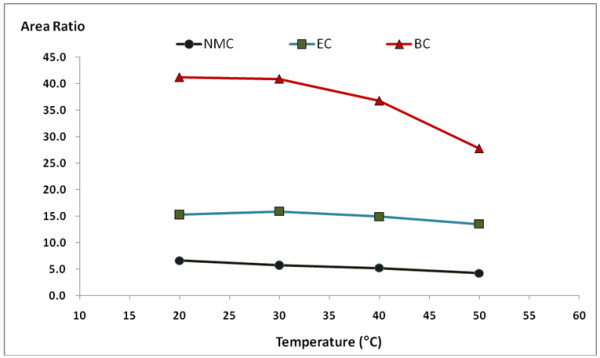
**Effect of reaction temperature on the reaction of MC, EC and BC with 9-xanthydrol.** (This experiment was performed at a reaction time of 5 min).

**Figure 5:**
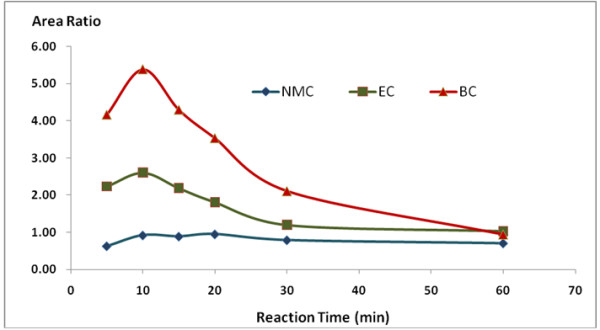
**Effect of reaction time on the reaction of MC, EC and BC with 9-xanthydrol.** (This experiment was performed at a reaction temperature of 20°C).

The optimal reaction conditions for the simultaneous determination of MC and EC in solid fermented foods was also tested. For the first test, the minimum amount of 9-xanthydrol for the derivatization was studied. The derivatization was performed for various 9-xanthydrol concentrations (1.0, 2.0, 3.0, 4.0, 5.0 and 6.0 mM of 9-xanthydrol). The yield stayed continuously beyond 4.0 mM of 9-xanthydrol and the optimal 9-xanthydrol amount was 4.0 mM (Figure 2). The effect of the acid concentration on the reaction of MC, EC and BC with 9-xanthydrol was also studied. The derivative was tested at HCl concentrations of 0.01, 0.05, 0.1, 0.2, 0.3, and 0.5 M. The other reaction conditions were set to have a reaction time of 10 min at a temperature of 20°C. The results showed good recovery at the HCl concentration value of 0.2 M (Figure 3). The reaction rate of MC, EC and BC with 9-xanthydrol was also studied. The reaction rate of the derivative was analyzed at reaction temperatures of 20, 30, 40, and 50°C and the reaction time was analyzed in at 5, 10, 20, 30, and 60 min. From the experiment, the optimal reaction temperature and time was 10 min at 20°C (Figures 4 and 5). The recovery was declined slowly beyond the reaction time of 10 min.

As a result, the optimal reaction conditions of MC, EC, and BC with 9-xanthydrol were 4.0 mM 9-xanthydrol, 0.2 M HCl concentration, the reaction time of 10 min at an ambient temperature.

The selection of the extraction solvent was of great importance in order to achieve satisfactory extraction efficiency for the target compounds. Based on the consideration for the solvent strength, methylene chloride, ethyl acetate, ethyl ether and hexane were selected as potential extraction solvents for use in this study. As a result, ethyl acetate gave the highest extraction efficiency, and ethyl acetate was selected as an extraction solvent of the analyte derivatives from samples.

### Chromatography and mass spectrometry

**Figure 6:**
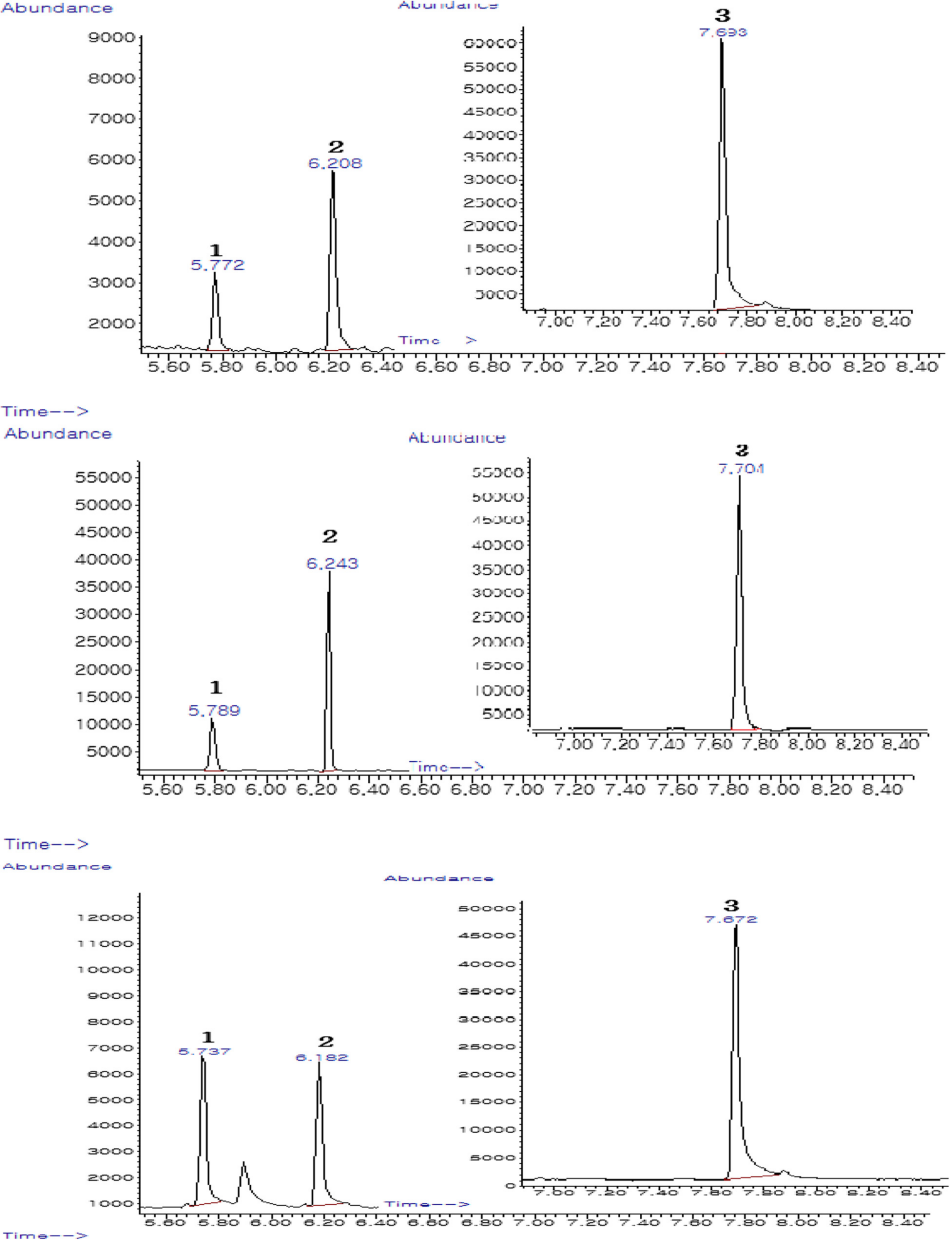
**SIM chromatograms after the derivatization of NMC, EC and BC from spiked food (top=25 μg/kg, middle=5 μg/kg) and real sample (bottom).** 1=Xanthyl methylcarbamate, 2=Xanthyl ethylcarbamate, 3=Xanthyl butylcarbamate (ISTD).

The optimum derivatization conditions were applied to the analysis of MC, EC, and BC in fermented food and beverages by GC-MS. Figure 6 shows the GC-MS chromatogram after the derivatization of MC, EC, and BC. For the GC separation of the derivative, the use of a nonpolar stationary phase was found to be efficient. The derivatives of MC, EC, and BC showed a sharp peak, and the compound was quantified as an integration of the peak area. The retention times of xanthyl methylcarbamate, xanthyl ethylcarbamate and xanthyl butylcarbamate are shown in Figure 6. Extraneous peaks were not observed in the chromatograms near the retention times of the analytes.

**Figure 7:**
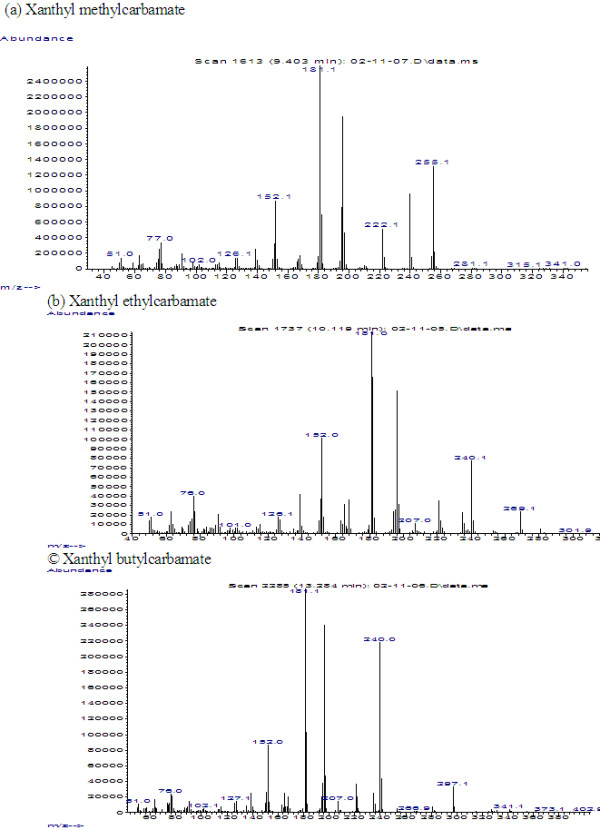
Mass spectra of xanthyl methylcarbamate, xanthyl ethylcarbamate and xanthyl butylcarbamate.

The mass spectra of xanthyl methylcarbamate, xanthyl ethylcarbamate and xanthyl butylcarbamate by electron ionization at 70 eV have similar fragmentation pattern as shown in Figure 7. The molecular ions at m/z 255, m/z 269 and m/z 297 were appeared in mass spectra of three compounds. The fragment of m/z 240 was accounted for by the loss of [CH_3_], [C_2_H_5_] and [C_4_H_9_] from the each molecular ion and that of m/z 196 was accounted for by the loss of [COOCH_3_], [COOC_2_H_5_] and [COOC_4_H_9_], and m/z 222 were accounted for by the loss of [H_2_OCH_3_], [H_2_OC_2_H_5_] and [H_2_OC_4_H_9_] from the each molecular ion. The fragment of m/z 181 was a result of the xanthyl group.

### Validation of the assay

The combination of a high derivatization yield and the high sensitivity of the derivative by EI-MS (SIM) allowed the detection of MC and EC at concentrations well below those reported previously. The limit of detection (LOD) and the limit of quantification (LOQ) were defined as the analyte concentration corresponding to a signal/noise ratio of 3 and 10 in the control food, in which MC and EC were not detected. The LODs in this study were 0.11 μg/kg for MC, and 0.12 μg/kg for EC, and the LOQs were 0.35 μg/kg for MC and 0.38 μg/kg for EC using a 2.0 g sample. Table 1 compares various analytical methods for determining the MC and EC in fermented food and beverages. The method permits the determination of two analytes below that detected previously using the GC-MS method, which was otherwise slightly higher than GC-HRMS or GC-MS/MS methods.

**Table 1:** Comparison of analytical methods for determining of NMC and EC in fermented food

**Reference**	**Matrix**	**Preparation method**	**Derivatization**	**Measurement**	**LOD(μg/L or μg/kg)**		**LOQ (μg/L or μg/kg)**	
					**MC**	**EC**	**MC**	**EC**
[10]	Alcoholic beverage	-	9-xanthydrol	HPLC-FLD		3.0-	-	-
[11]	Wine	-	9-xanthydrol	HPLC-FLD	-	73.2-	-	243.9-
[12]	Cider spirits	-	9-xanthydrol	HPLC-FLD	-	1.64	-	3.56
[13]	Ethanol solution	-	-	LC-MS/MS		2.0	-	5.1
	Palinka spirits			LC-MS/MS				
						2.8	-	8.0
[15]	Alcoholic beverage	HS-SPME	-	GC-NPD	-	34	-	-
[19]	Alcoholic beverage	LLE	-	GC-MS	-	2.3	-	10.4
[20]	Fermented food	SPE	-	GC-HRMS	-	0.03	-	0.05
[22]	Stone-fruit spirits	HS-SPME	-	GC-MS/MS	-	0.03	-	0.11
[21]	Stone-fruit spirits	SPE	-	GC-MS/MS	-	0.01	-	0.04
[27]	Italian aqua vitae	LLE	9-xanthydrol	GC-MS	-	1.0	-	-
This study	Fermented food and beverage	LLE	9-xanthydrol	GC-MS	0.11	0.12	0.33	0.38

The calibration curves of the MC and EC were constructed by the reaction and extraction of the spiked food samples. Examination of the standard curve by computing a regression line of the peak area ratios for the MC and EC to the internal standard on concentrations using a least-squares fit demonstrated a linear relationship with correlation coefficients of 0.998 and 0.996, respectively. The line of best fit for the MC was y = 4.191 x - 0.0001 over a range of 1.0-100 μg/kg and that for EC was y = 13.46 x + 0.0051 over a range of 1.0-100 μg/kg, where x is the analyte concentration (mg/kg) and y is the peak area ratio of the analyte to the internal standard.

The accuracy can be assessed by determining the recovery in spiked samples: Intra-day accuracy was evaluated using five spiked samples at concentrations of 0.05 and 0.002 μg/kg for MC and EC, respectively. The inter-day accuracy was determined using the sample recovery on three different days. The accuracy was in range of approximately 90- to 109% and the precision of the assay was less than 12%, as shown in Table 2.

**Table 2:** **Intraday and interday laboratory precision and accuracy results for the analysis of NMC and EC in fermented food** (***n***=**5**)

**Compound**	**Spiked Conc**.**(mg/L)**	**Intraday measured value**			**Interday measured value**		
		**Mean±SD(mg/L)**	**Accuracy (%)**	**Precision (%)**	**Mean±SD(mg/L)**	**Accuracy (%)**	**Precision (%)**
NMC	0.0500	0.0492 ± 0.0031	98.4	6.30	0.0474 ± 0.0052	94.8	11.0
	0.0020	0.0018 ± 0.0002	90.0	11.1	0.0017 ± 0.0002	106	11.8
EC	0.0500	0.0532 ± 0.0030	106	5.64	0.0546 ± 0.0065	109	11.9
	0.0020	0.0018 ± 0.0002	90.0	11.1	0.0019 ± 0.0002	95.0	10.5

### Food analysis

This paper was designed to describe a method to detect MC and EC in solid and liquid state matrices using GC-MS. Generally, many traditional Korean foods are made through fermentation of a mixture of various food materials, and therefore these foods have complicated matrix properties. When the proposed method was applied to the food items, interfering peaks were not observed in the chromatograms near the retention times of the analytes.

Using the proposed method, the levels of MC and EC were analyzed in sixteen traditional fermented Korean foods, including soybean paste, red pepper paste, and soy sauce, and eleven beverages and the results were shown in Table 3. MC was detected in a range from 0.4 to 0.8 μg/L in mainly fruit liquors. Most samples had detectable levels of EC in a range from 0.4 to 85.8 μg/L or μg/kg. The concentration range of the EC of each food or beverage type was found for soybean paste (0.9-2.7 μg/kg), red pepper paste (0.7-2.3 μg/kg), soy sauce (0.4-8.9 μg/L), and beverages (not detected-85.8 μg/L). From the results shown in Table 3, the prolonged mean storage time had no relationship with the detected content of EC.

**Table 3:** Analytical results of the NMC and EC in fermented food and beverages

**Sample**	**State**	**Storage time(yr)**	**Unit**	**Measured Conc(μg/kg)**	
				**MC**	**EC**
Red pepper paste-1	Solid	4	μg kg^-1^	nd	0.7
Red pepper paste-2	Solid	3	μg kg^-1^	nd	1.3
Red pepper paste-3	Solid	3	μg kg^-1^	nd	1.9
Red pepper paste-4	Solid	2	μg kg^-1^	nd	1.8
Red pepper paste-5	Solid	1	μg kg^-1^	nd	2.3
Red pepper paste-6	Solid	1	μg kg^-1^	nd	0.9
Soybean paste-1	Solid	4	μg kg^-1^	nd	0.9
Soybean paste-2	Solid	3	μg kg^-1^	nd	1.5
Soybean paste-3	Solid	2	μg kg^-1^	nd	1.2
Soybean paste-4	Solid	4	μg kg^-1^	nd	1.7
Soy sauce-1	Liquid	2	μg L^-1^	nd	1.3
Soy sauce-2	Liquid	1	μg L^-1^	nd	0.4
Soy sauce-3	Liquid	1	μg L^-1^	0.4	8.9
Soy sauce-4	Liquid	1	μg L^-1^	nd	1.8
Soy sauce-5	Liquid	1	μg L^-1^	nd	0.8
Soy sauce-6	Liquid	1	μg L^-1^	nd	1.3
Beer	Liquid	-	μg L^-1^	nd	3.9
Soju(white distilled liquor)	Liquid	-	μg L^-1^	nd	4.8
Jeongjong(refined rice wine)	Liquid	-	μg L^-1^	0.5	8.3
Soju(distilled liquor)	Liquid	-	μg L^-1^	nd	nd
Makgeolli(raw rice wine)-1	Liquid	-	μg L^-1^	nd	nd
Makgeolli(raw rice wine)-2	Liquid	-	μg L^-1^	0.5	6.9
Makgeolli(raw rice wine)-3	Liquid	-	μg L^-1^	nd	6.0
Fruit liquor-1	Liquid	-	μg L^-1^	nd	4.1
Fruit liquor-2	Liquid	-	μg L^-1^	0.6	78.7
Fruit liquor-3	Liquid	-	μg L^-1^	0.7	68.6
Fruit liquor-4	Liquid	-	μg L^-1^	0.8	85.8

The correlations between the levels of EC and MC in beverages also correlated well with each another (r^2^=0.69, P=0.001) due to the similar formation mechanisms. It is suggested that MC is also formed by the reaction of urea with methanol.

## Conclusions

In this paper, a simple, sensitive method to detect MC and EC in several solid foods and liquid foods is presented based on derivatization with 9-xanthydrol for 10 min at an ambient temperature. Using 2.0 g for solid food and liquid food, the LODs of the MC and EC were 0.11 and 0.12 μg/kg, respectively, and the LOQs of the MC and EC were 0.35 and 0.38 μg/kg, respectively. The accuracy and precision of the assay were acceptable: the relative standard deviation was less than 12%. The concentrations of MC and EC in Korean traditional fermented foods were measured to be to 85.8 μg/kg. The natural levels of MC and EC found in these foods are not considered to pose a risk to human health.

## Competing interests

The authors declare that they have no competing interests.

## Authors’ contributions

HSS initiated and prepared the draft. EYY conducted the extraction and method developments. All authors designed the study. All authors contributed to data analyses and to finalizing the manuscript. Both authors have read and approved the final version.
